# Sex differences in cachexia and branched‐chain amino acid metabolism following chemotherapy in mice

**DOI:** 10.14814/phy2.16003

**Published:** 2024-04-17

**Authors:** Stephen Mora, Gagandeep Mann, Olasunkanmi A. J. Adegoke

**Affiliations:** ^1^ Muscle Health Research Centre, School of Kinesiology and Health Science York University Toronto Ontario Canada

**Keywords:** branched‐chain amino acid, cachexia, chemotherapy, metabolism, sex

## Abstract

Chemotherapy is a major contributor to cachexia, but studies often investigate male animals. Here, we investigated whether sex modifies the effects of chemotherapy on cachexia and BCAA metabolism. Ten‐week‐old CD2F1 male and female mice were treated with the chemotherapy drug cocktail folfiri (50 mg/kg 5‐fluorouracil, 90 mg/kg leucovorin, and 24 mg/kg CPT11) (drug) or vehicle twice a week for 6 weeks. Insulin tolerance tests were conducted and BCAA levels and metabolism were measured in plasma and tissues. Drug treatment reduced body and skeletal muscle weights and anabolic signaling in both sexes, with females showing worsened outcomes (*p* < 0.05 for all). Drug treatment increased plasma BCAA only in males, but BCAA concentrations in the skeletal muscle of both sexes were decreased; this decrease was more profound in males (*p* = 0.0097). In addition, muscle expression of the BCAA transporter LAT1 was reduced; this reduction was more severe in females (*p* = 0.0264). In both sexes, the (inhibitory) phosphorylation of BCKD‐E1α^ser293^ was increased along with decreased BCKD activity. In the liver, drug treatment increased BCAA concentrations and LAT1 expression, but BCKD activity was suppressed in both sexes (*p* < 0.05 for all). Our results demonstrate that altered BCAA metabolism may contribute to chemotherapy‐induced cachexia in a sex‐dependent manner.

## Graphical abstract

Sex differences in chemotherapy‐induced cachexia are linked to altered BCAA handling.

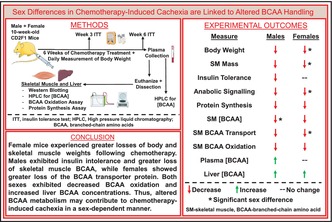

## 1 INTRODUCTION

Sex is a major risk factor for cancers (Kim et al., 2018). Affecting 20%–80% of cancer patients dependent on cancer stage and type is cachexia, a devastating body and skeletal muscle wasting syndrome (Lim et al., 2020). Systemic inflammation, insulin resistance, increases in energy expenditure, negative protein/energy balance, appetite loss, anorexia, and fatigue are all experienced in cachexia (Evans et al., 2008). Chemotherapy is also a major contributor to skeletal muscle loss and cachexia (Ballarò et al., 2019; Barreto, Waning, et al., 2016; Pin et al., 2019).

During cachexia, autophagy/lysosomal and ubiquitin proteasome pathways (UPP), two pathways implicated in skeletal muscle protein breakdown, are upregulated (Tisdale, 2005), leading to skeletal muscle wasting. Muscle protein synthesis is regulated by the mammalian/mechanistic target of rapamycin complex 1 (mTORC1). Upstream, mTORC1 is activated through the insulin receptor substrate‐1 (IRS‐1)/phosphatidylinositol‐3 kinase (PI3K)/protein kinase B (AKT) pathway which allows RHEB, an mTORC1 activator, to stay GTP loaded. mTORC1 sensing of amino acids (AAs) and especially the branched‐chain amino acids (BCAAs: leucine, isoleucine, and valine) is also required for full activation of the complex (Adegoke et al., 2012). Activation of components of the sestrins/gator/RAG/ragulator pathway by AA and BCAA leads to the translocation of mTORC1 to the lysosomal membrane where RHEB is localized (Chantranupong et al., 2014). Once activated, mTORC1 phosphorylates several downstream targets, such as ribosomal protein S6 kinase beta‐1 (S6K1) and eukaryotic translation initiation factor 4E‐binding protein 1 (4E‐BP1) leading to protein synthesis (Adegoke et al., 2012). Due to this, the BCAA are potent stimulators of skeletal muscle protein synthesis (Wolfe, 2017) and represent a promising target to treat cachexia, as the BCAA have shown some positive effects on reversing cancer‐induced cachexia (Gomes‐Marcondes et al., 2003; Ventrucci et al., 2004). However, nutritional interventions using the BCAA to mitigate cachexia have yielded minimal benefits in humans (Harima et al., 2010; Pimentel et al., 2021; Poon et al., 2004; Soares et al., 2020).

Apart from activating protein synthesis, the BCAA can also be metabolized in the skeletal muscle and other organs/tissues, including the liver and adipose tissue. Branched‐chain aminotransferase (BCAT2) reversibly transaminates the BCAA into their respective branched‐chain α‐keto acids (BCKA: 2‐keto‐isocaproate/4‐methyl‐2‐oxopentanoic acid (KIC) from leucine, α‐keto‐β‐methylvaleric acid/3‐methyl‐2‐oxopentanoate (KMV) from isoleucine, and 2‐keto‐isovalerate/3‐methyl‐2‐oxobutanoic acid (KIV) from valine) and glutamate. The branched‐chain α‐keto acid dehydrogenase complex (BCKD) then oxidatively decarboxylates the BCKA producing their corresponding acylCoA derivatives: isovaleryl‐CoA from KIC, 2‐methylbutyryl‐CoA from KMV, and isobutyryl‐CoA from KIV (Mann et al., 2021). Levels of enzymes involved in BCAA metabolism, including BCAT1/2 and BCKD, are increased in cancers of the liver (Ericksen et al., 2019) and breast (Zhang & Han, 2017). However, the impact of cancer and/or chemotherapy on the metabolism of the BCAA in the skeletal muscle is rarely studied. Therefore, studying BCAA metabolism in the skeletal muscle following chemotherapy may provide insights into the inability for BCAA to successfully treat cachexia.

Many of the previous studies on chemotherapy‐induced cachexia are conducted in male animals (Barreto, Mandili, et al., 2016; Barreto, Waning, et al., 2016; Pin et al., 2019; Rosa‐Caldwell & Greene, 2019). However, there is evidence for sex differences in BCAA metabolism. Compared to females, males have higher concentrations of the BCAA (Costanzo et al., 2022) and greater leucine oxidation after endurance exercise (Mittendorfer et al., 2002). Further, estrogen decreases BCKD activity, but this study only investigated female animals (Obayashi et al., 2004). Here, we hypothesized that chemotherapy would induce greater catabolism of BCAA in tissues of male mice, and that this change would be associated with more severe cachexia.

## 2 MATERIALS AND METHODS

### 2.1 Animals

Fourteen male and 14 female 8‐week‐old CD2F1 were purchased from Charles River and Envigo Laboratories. Because of supply issues from our original supplier, female mice of the same strain and at similar age were obtained from Envigo. Mice were acclimatized and housed in the vivarium with free access to food (Purina 5015*, LabDiet, St. Louis, MO) and water. All animals were aged until 10 weeks before treatment. For females, estrous tracking occurred 1‐week prior to treatment to ensure that female mice were in the same stage of their estrous cycle. Chemotherapy treatment began when female mice were in di‐estrous. Mice were separated into four groups: male vehicle, male drug, female vehicle, female drug (*n* = 7 for all groups). Mice were administered intraperitoneally (i.p.) either the chemotherapy drug cocktail folfiri (50 mg/kg 5FU (#F6627), 90 mg/kg Leucovorin (#F7878) and 24 mg/kg CPT11 (#I1406)) (drug) or vehicle ((3.8% DMSO (#D5879), all from Sigma Aldrich, St. Louis, MO) in saline) twice a week for 6 weeks. The two weekly doses were separated by 2 days. This drug cocktail is typically used in the treatment of colorectal cancer (Tabernero et al., 2015). Drug dosages were taken from a previous study whereby this cocktail induced atrophy in the skeletal muscle of mice (Barreto, Mandili, et al., 2016). Bodyweight and food consumption were recorded daily. However, to account for minor differences in baseline values, body weight was presented as weekly variations relative to Day 0 as presented by Barreto et al. (2016). At the end of the study, animals were euthanized via cervical dislocation at least 24 h after the last chemotherapy dose. Following sacrifice, the gastrocnemius, tibialis anterior and quadriceps were collected. Visceral white adipose tissue, kidney, liver, and spleen were also collected. Tissues were weighed, flash‐frozen in liquid nitrogen and stored at −80°C until analysis. The gastrocnemius muscle was used for the subsequent analyses, as a sex difference was observed in the weight of this muscle following treatment.

### 2.2 Insulin tolerance test

At both the third and sixth weeks of treatment, insulin tolerance tests (ITT) were performed. With at least 24 h after their previous chemotherapy dose, mice were fasted for 6 h and 0.75 units/kg of insulin (Eli Lilly, Humulin R, #00586714, Indianapolis, IN) was administered via subcutaneous injection. Blood samples were collected from the saphenous vein on glucose strips (Alpha TRAK, #71681, Parsippany, NJ) at 0 (baseline), 5, 15, 30, and 120 min post insulin injection. The strips were inserted into a glucometer (Alpha TRAK, #71675‐01) to obtain glucose concentrations.

### 2.3 BCKD activity assay

We measured BCKD activity using a modified version of a previous assay (White et al., 2018). Approximately 30 mg of frozen gastrocnemius muscle or liver were crushed in liquid nitrogen and homogenized (Bio‐Gen PRO200 Homogenizer, PRO Scientific, Oxford, CT) in 250 μL of ice‐cold buffer 1 (30 mM potassium phosphate buffer (KPI), 3 mM EDTA, 5 mM DTT, 1 mM valine, 3% FBS, 5% Triton X‐100 and 1 μm leupeptin (Sigma Aldrich, #L2884)). The resulting sample was centrifuged (10 min at 10,000 g, 4°C) and 50 μL of the supernatant was added to 300 μL of buffer 2 (50 mM HEPES, 30 mM KPI, 0.4 mM CoA (Sigma Aldrich, #C4282), 3 mM NAD+ (Sigma Aldrich, #N0632), 5% FBS, 2 mM Thiamine (Sigma Aldrich, #T1270), 2 mM magnesium chloride and 7.8 μM [^14^C] valine (Perkin Elmer, #NEC291EU050UC, Waltham, MA)). This reaction took place in a 1.5‐mL Eppendorf tube containing a raised 2‐M NaOH wick trap. Each Eppendorf tube was sealed tight and placed in a shaking incubator at 37°C for 30 min. The radiolabeled ^14^CO_2_ contained in the wick trap was counted in a liquid scintillation counter.

### 2.4 Western blotting

The procedures used have been described (Mann & Adegoke, 2021). Briefly, tissue samples were homogenized in 7X complete buffer: 20 mM HEPES, 2 mM EGTA, 50 mM NaF, 100 mM KCI, 0.2 mM EDTA, and 50 mM B‐Glycerophosphate, supplemented just before use with 10 μL/mL of each of protease inhibitor (Sigma Aldrich, #P8340) and phosphatase inhibitor cocktails (Sigma Aldrich, #P5726), 0.2 M sodium vanadate (2.5 μL/mL), 1 M DTT (1 μL/mL) and 0.2 M benzamidine (5 μL/mL). Homogenates were then centrifuged at 1000 g for 3 min at 4°C. The resulting supernatant was removed and centrifuged again at 10,000 g for 30 min at 4°C. Protein concentrations in the supernatant were determined using the Pierce BCA Protein Assay Kit (Thermo Scientific, #23225, Waltham, MA). Equal amounts of protein (~25 μg) were separated on 10% or 15% SDS‐PAGE gels and transferred onto polyvinylidene difluoride (PVDF) membranes (0.2 μM, BIO‐RAD). Incubation of membranes in primary (Table S1) and secondary antibodies (HRP‐conjugated anti‐rabbit (#7074) or anti‐mouse (#7076), Cell Signaling Technology, Danvers, MA), imaging and quantification of data were as described (Jeganathan et al., 2014; Mora & Adegoke, 2021; Zargar et al., 2011).

### 2.5 High pressure liquid chromatography (HPLC)

This was done as previously described (Mann & Adegoke, 2021). Briefly, skeletal muscle and liver samples were homogenized and centrifuged. AAs present in the supernatant were then diluted, pre‐column derivatized in a ratio of 1 (sample): 1 (o‐phthalaldehyde, Sigma Aldrich, #P1378), and injected into a YMC‐Triart C18 column (C18, 1.9 μm, 75 × 3.0 mm; YMC America, Allentown, PA, USA) fitted onto an ultra HPLC system (Nexera X2, Shimadzu, Kyoto, Japan) connected to a fluorescence detector (Shimadzu, Kyoto, Japan; excitation: 340 nm; emission: 455 nm). Plasma samples were similarly diluted, pre‐column derivatized and analyzed. AA standard (Sigma Aldrich, #AAS18) curves were used to calculate AA concentrations, and values in skeletal muscle and liver samples were normalized to total protein.

### 2.6 Protein synthesis (SUnSET analysis)

With at least 24 h after their last chemotherapy dose, mice were starved for 3 h in order to limit the effects of nutrient uptake on protein synthesis. Thirty minutes prior to euthanasia, mice were intraperitoneally injected with 0.040 μmol/g bodyweight of puromycin (Sigma Aldrich, #P8833) in saline. Skeletal muscles were removed after euthanasia and muscle proteins were immunoblotted against an anti‐puromycin antibody and corrected to their Ponceau S staining.

### 2.7 Statistics

All immunoblot analyses were quantified and adjusted to their corresponding γ‐tubulin values. Graphs were drawn using Prism 10 (GraphPad software). Because male and female mice were obtained from separate vendors, we used unpaired *t*‐tests to measure treatment differences within each sex. We calculated percentage changes due to treatment (within each sex) and then used an unpaired *t*‐test to compare whether those treatment‐induced percentage changes differed between the sexes. For the ITT data, within sex, a two‐way‐ANOVA was used to measure blood glucose differences following chemotherapy across all time points, followed by area under the curve (AUC) analysis. We also measured and compared the slopes of the lines between the 5 and 15 min time points in drug‐treated male and female mice at 6 weeks. Results were expressed as mean ± standard deviation (SD). Significance was determined as *p* < 0.05.

## 3 RESULTS

### 3.1 Drug‐treated female mice have worsened outcomes for body and skeletal muscle weight following chemotherapy

**FIGURE 1:**
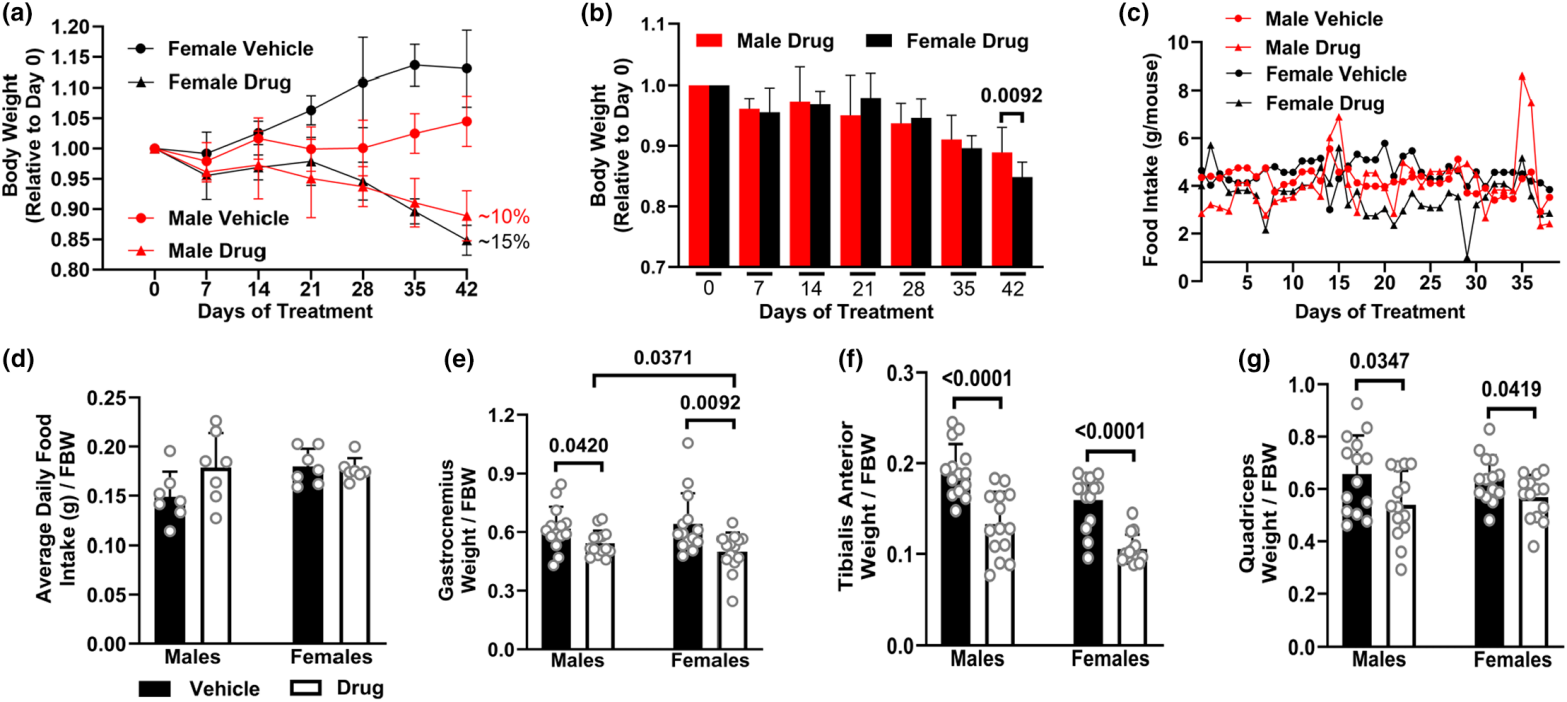
Drug‐treated female mice have worsened outcomes for body and skeletal muscle weight following chemotherapy. Male and female 10‐12‐week‐old CD2F1 mice were treated with either vehicle (V, 3.8% DMSO in saline, black bars) or a chemotherapy drug cocktail (D, Drug: 50 mg/kg 5‐FLU, 90 mg/kg Leucovorin, 24 mg/kg CPT11, white bars) twice a week for 6 weeks. Body weight (a, b) and food intake (c, d) were recorded daily for 6 weeks. Animals were euthanized at least 24 h after their last chemotherapy dose. Weights of the skeletal muscles, corrected for final body weight (FBW): gastrocnemius (e), tibialis anterior (f) and quadriceps (g). Data are mean ± SD; n = 7 animals per group.

Following 6 weeks of chemotherapy treatment, drug‐treated males lost ~10% body weight, while drug‐treated females lost ~15% (relative to their baseline weight, Figure 1a; raw body and tissue weights are shown in Table S2). At Week 6, body weight loss was higher in female than in male (Figure 1b). Drug treatment did not affect food intake (Figure 1c,d). The loss of body weight in drug‐treated animals was consistent with significant decreases in the weights of the gastrocnemius (Figure 1e), tibialis anterior (Figure 1f) and quadriceps (Figure 1g) muscle. The loss of gastrocnemius muscle weight in females was more severe compared to males (Figure 1e). There were no effects of drug treatment on adipose tissue and kidney weights, but both sexes showed a significant increase in spleen weight, while only drug‐treated females showed an increase in liver weight compared to controls (Figure S1A–D). Because of the sex difference in response to chemotherapy in the gastrocnemius muscle weight, all the subsequent muscle‐based measures were done in this muscle. Protein expression of gastrocnemius muscle contractile proteins MHC‐1, troponin, and tropomyosin were decreased in both sexes following chemotherapy (Figure S1E–H).

### 3.2 Chemotherapy causes greater insulin resistance in males, but drug‐treated female mice experience worsened outcomes for anabolic and catabolic signaling

**FIGURE 2:**
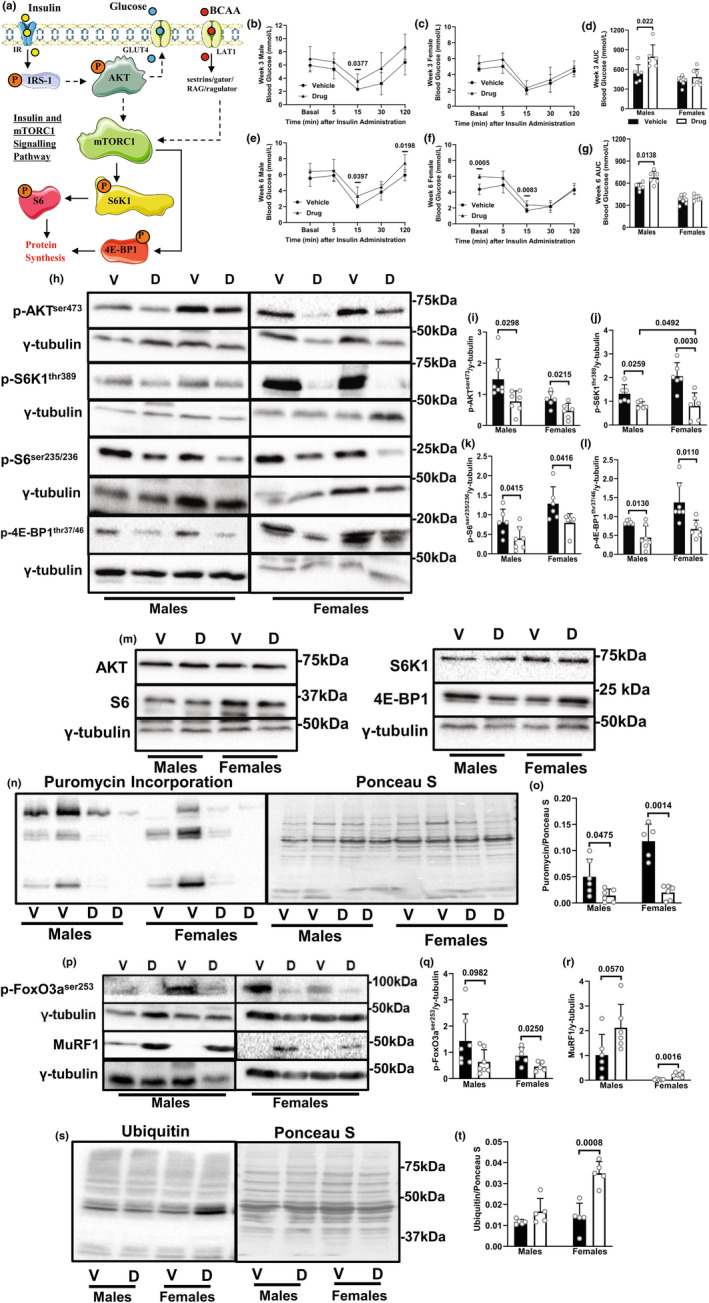
Chemotherapy causes greater insulin resistance in males, but drug‐treated female mice experience worsened outcomes for anabolic and catabolic signaling. Animals were treated as described in Figure 1. (a) Insulin binds to the insulin receptor (IR) leading to the phosphorylation of IRS‐1 on tyrosine residues and the eventual activation of AKT. AKT activation leads to an increase in insulin stimulated glucose uptake in skeletal muscle. AKT also indirectly activates mTORC1, leading to the phosphorylation of S6K1 and 4E‐BP1, ultimately leading to an increase in protein synthesis. The BCAA, transported into skeletal muscle through LAT1, activate components in the sestrins/gator/RAG/ragulator pathway, leading to the activation of mTORC1. During Weeks 3 and 6 of treatment, with at least 24 h after their previous chemotherapy dose, male (b, e) and female (c, f) mice were starved for 6 h and then underwent an ITT. Blood glucose AUC in both sexes is shown (d, g). Immunoblotting and quantified data for p‐AKT^ser473^ (h, i), p‐S6K1^thr389^ (h, j), p‐S6^ser235/236^ (h, k) and p‐4E‐BP1^thr37/46^ (h, l) in the gastrocnemius muscle are shown. Immunoblots are shown for total AKT, S6, S6K1 and 4E‐BP1 in the gastrocnemius (m). Protein synthesis was measured via the SUnSET analysis at least 24 h from their previous chemotherapy dose. Thirty minutes prior to euthanasia, mice were injected with 0.040 μmol/g bodyweight of puromycin. Following euthanasia, tissues were isolated. Skeletal muscle proteins were immunoblotted against an anti‐puromycin antibody and corrected to their respective Ponceau S staining (n, o). Immunoblotting and quantified data for p‐FoxO3a^ser253^ (p, q), MuRF1 (p, r), and ubiquitinated proteins (s, t) in the gastrocnemius muscle are shown. Data are mean ± SD; *n* = 5–7 animals per group.

A simplified diagram of insulin, BCAA and mTORC1 signaling is shown (Figure 2a). At Weeks 3 and 6 of treatment, male (Figure 2b,e), but not female (Figure 2c,f) drug‐treated mice showed impaired insulin tolerance, corresponding with higher blood glucose AUC (Figure 2d,g). Because the observed sex differences during the ITT could be due to higher basal (pre‐insulin injection) glucose levels in drug‐treated animals, we also calculated the slopes of the glucose curves between time points 5 and 15 min and found no differences at 6 weeks of chemotherapy in either sex (mean slope for male: −0.3217, mean slope for female: −0.3386, *p* = 0.7052).

In the gastrocnemius muscle, there was a decrease in the phosphorylation of AKT^ser473^, S6K1^thr389^, S6^ser235/236^ and 4E‐BP1^thr37/46^ compared to controls (Figure 2h,i). The decrease in phosphorylation of S6K1^thr389^ in drug‐treated females was more severe compared to males (Figure 2h,j). Total proteins of these phosphorylated targets were not affected by treatment (Figure 2m). Protein synthesis measured by SUnSET analysis was also decreased following drug treatment in both sexes (Figure 2n,o). For catabolic signaling, only drug‐treated female mice showed decreases in p‐FoxO3a^ser253^ (Figure P, Q), consistent with a treatment‐induced increase in MuRF1 expression (Figure 2p,r) and ubiquitinated proteins in females (Figure 2s,t).

### 3.3 Following chemotherapy, the reduction in skeletal muscle BCAA concentrations is more severe in males

**FIGURE 3:**
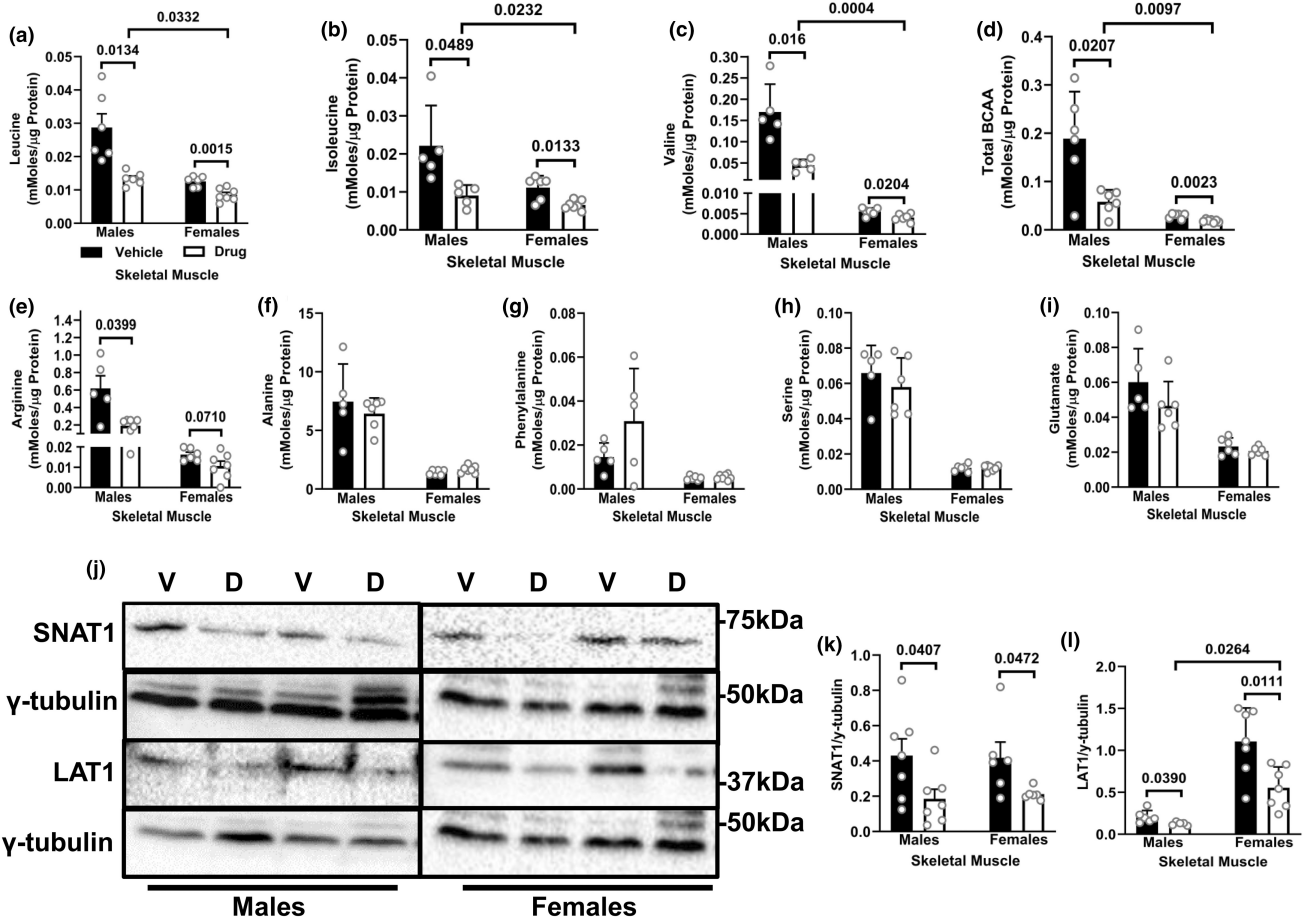
Following chemotherapy, the reduction in skeletal muscle BCAA concentrations is more severe in males. Animals were treated as described in Figure 1. Concentrations of leucine (a), isoleucine (b), valine (c), total BCAA (d), arginine (e), alanine (f), phenylalanine (g), serine (h) and glutamate (i) in the gastrocnemius muscle were measured by HPLC. Immunoblotting and quantified data for SNAT1 (j, k) and LAT1 (j, i) in the gastrocnemius muscle. Data are mean ± SD; *n* = 6–7 animals per group.

Concentrations of leucine, isoleucine, valine and total BCAA were reduced in drug‐treated animals (Figure 3a–d). These decreases were more severe in males. Only drug‐treated males showed a decrease in arginine concentrations (Figure 3e), while minimal differences were found for concentrations of alanine, phenylalanine, serine and glutamate (Figure 3f–i). Both sexes showed decreases in the expression of AA transporters SNAT1 (Figure 3j,k) and LAT1 (Figure 3j,l) following chemotherapy; however, the decrease in the expression of LAT1 was more severe in females than in males (Figure 3j,l).

### 3.4 Skeletal muscle BCAA catabolism is decreased following chemotherapy in both sexes

**FIGURE 4:**
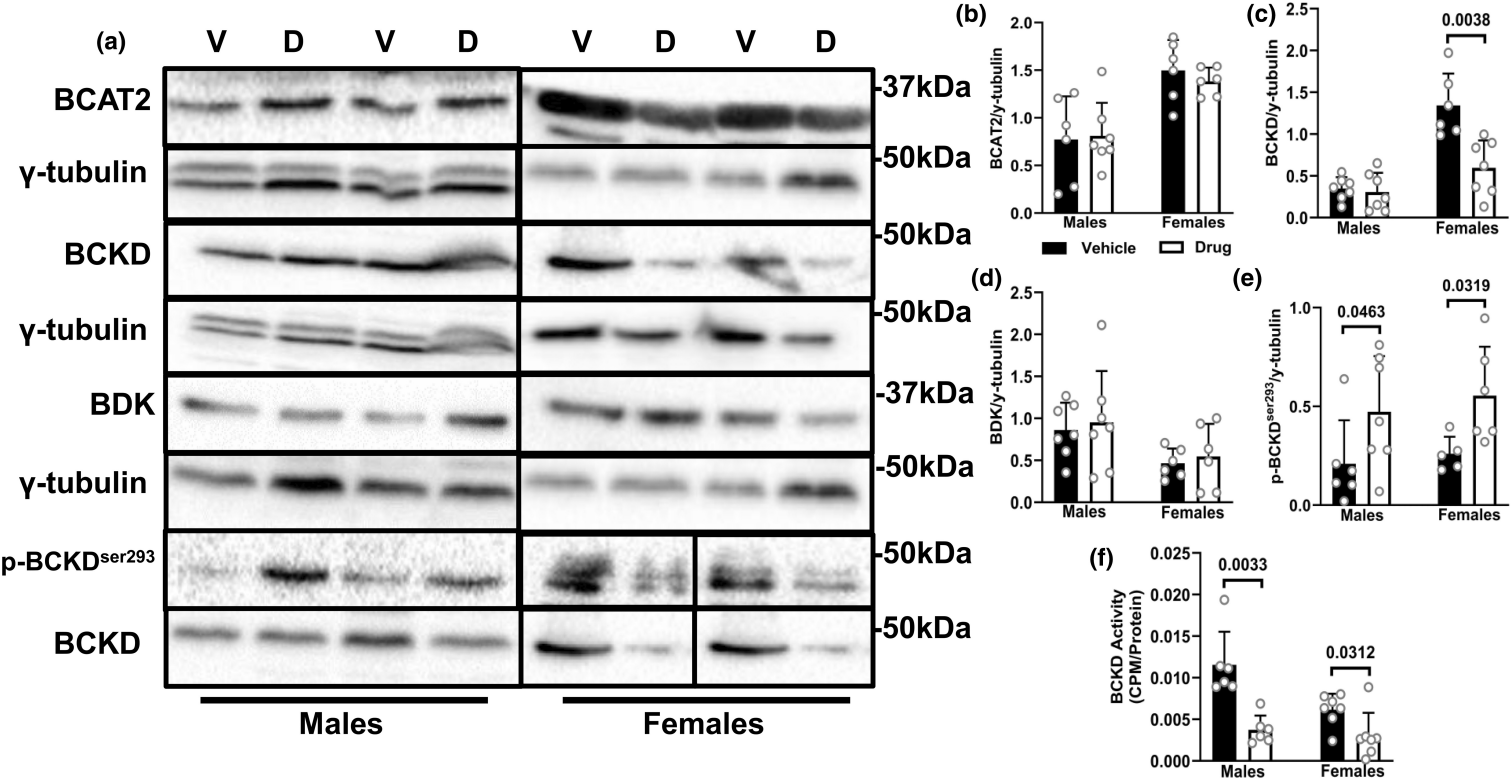
Skeletal muscle BCAA catabolism is decreased following chemotherapy in both sexes. Animals were treated as described in Figure 1. Immunoblotting (a) and quantified data for BCAT2 (b), BCKD‐E1α (c), BDK (d) and p‐BCKD‐E1α^ser293^ (e) in the gastrocnemius muscle. BCKD activity was measured from the release of ^14^CO_2_ from ^14^C labeled valine (f). Data are mean ± SD; *n* = 5–7 animals per group.

A simplified diagram of the first two steps in BCAA catabolism is shown (Figure S2). Protein expression of BCAT2 was unchanged following drug treatment (Figure 4a,b). Expression of BCKD‐E1α was unchanged in males, but decreased in drug‐treated female animals (Figure 4a,c). BDK was unchanged following drug treatment (Figure 4a,d). However, the inhibitory phosphorylation of BCKD‐E1α^ser293^ was increased in both sexes (Figure 4a,e), corresponding with decreased activity of this dehydrogenase complex (Figure 4f).

### 3.5 Chemotherapy treatment leads to elevated concentrations of liver BCAA in both sexes, but only males show elevated plasma BCAA

**FIGURE 5:**
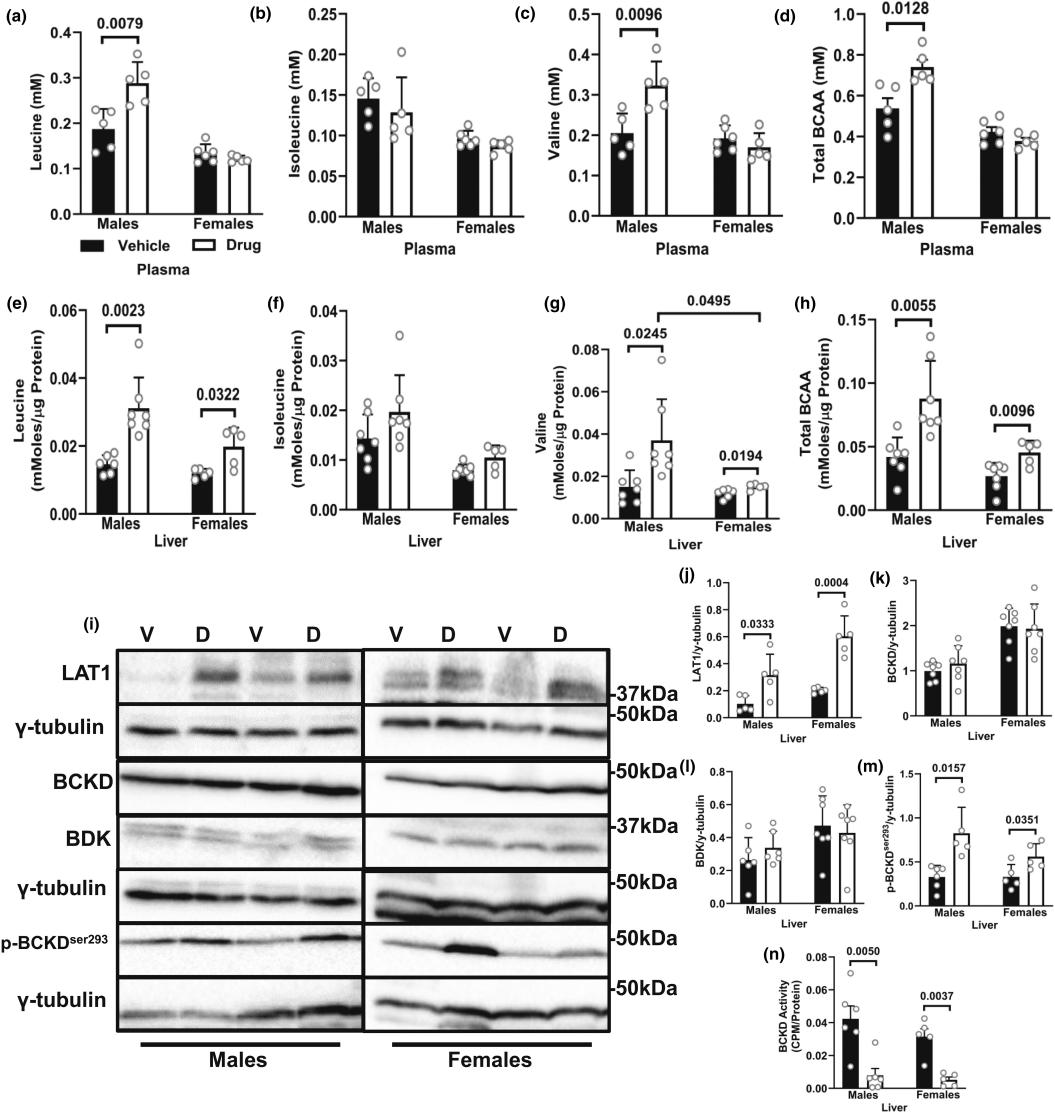
Chemotherapy treatment leads to elevated concentrations of liver BCAA in both sexes, but only males show elevated plasma BCAA. Animals were treated as described in Figure 1. Plasma leucine (a), isoleucine (b), valine (c) and total BCAA (d), as well as liver leucine (e), isoleucine (f), valine (g) and total BCAA (h) were measured by HPLC. Immunoblotting and quantified data of LAT1 (i, j), BCKD‐E1α (i, k), BDK (i, l) and p‐BCKD‐E1α^ser293^ (i, m) in the liver. BCKD activity in the liver (n). Data are mean ± SD; *n* = 5–7 animals per group.

Only drug‐treated males showed elevated plasma concentrations of leucine, valine (but not isoleucine) and total BCAA (Figure 5a–d). We also found elevated concentrations of leucine, valine (but not isoleucine) and total BCAA (Figure 5e–h) in the livers of drug‐treated mice from both sexes, with males showing a greater increase in valine (Figure 5g). LAT1 expression was increased in the liver from both sexes (Figure 5i,j). There were no significant treatment effects on the protein expression of BCKD‐E1α and BDK (Figure 5i,k,l). However, similar to skeletal muscle, the inhibitory phosphorylation of BCKD‐E1α^ser293^ was higher following drug treatment (Figure 5i,m), corresponding with decreased activity of BCKD in the liver (Figure 5n).

### 3.6 The BCAA are positively correlated with gastrocnemius muscle weight, LAT1 transporter expression and BCKD activity

**FIGURE 6:**
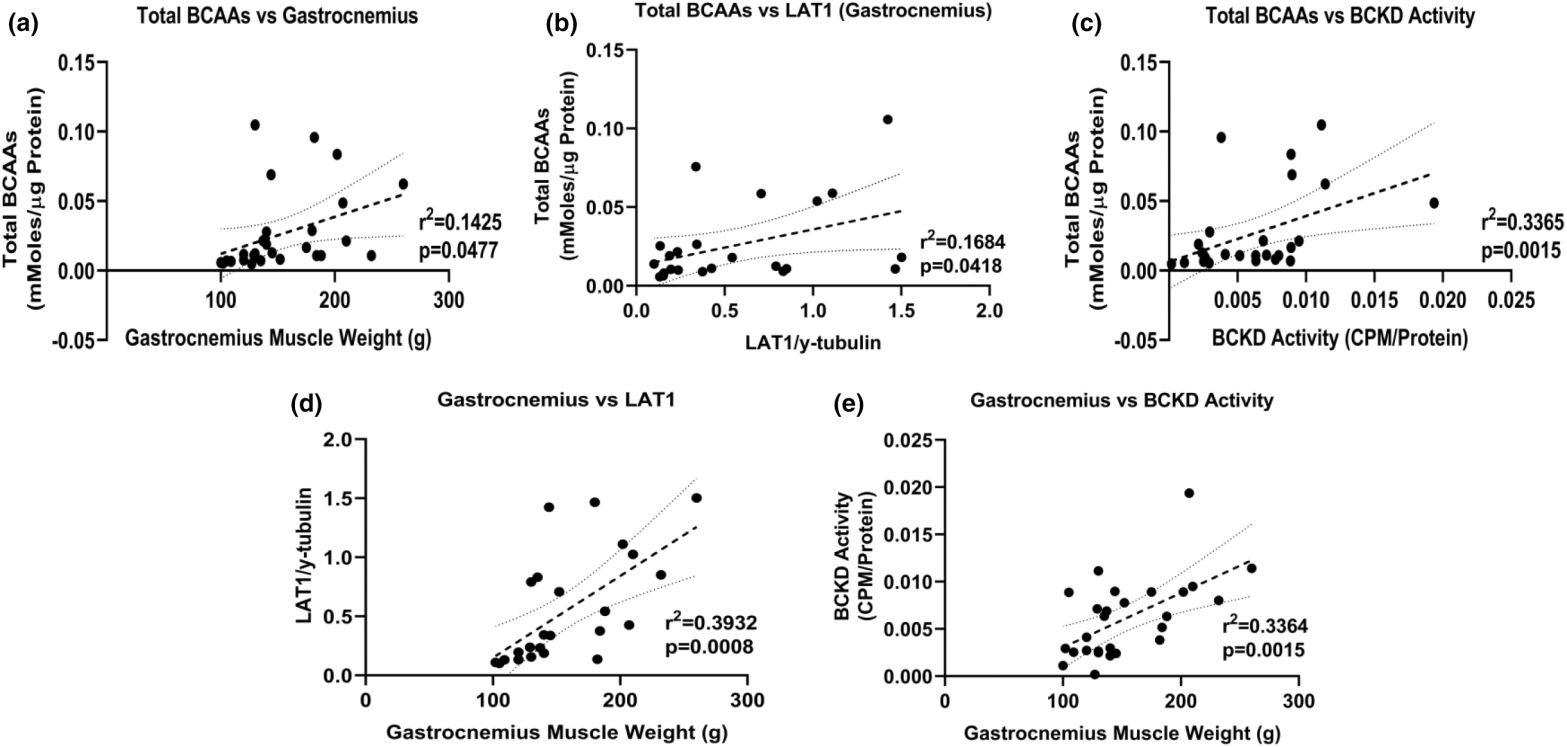
The BCAA are positively correlated with gastrocnemius muscle weight, LAT1 transporter expression and BCKD activity. Correlations between the BCAA and gastrocnemius muscle weight (a), skeletal muscle LAT1 (BCAA transporter) expression (b) and skeletal muscle BCKD activity (c). Correlations between gastrocnemius muscle weight and LAT1 (d) and BCKD activity (e) are also shown. Data were analyzed using linear regression and 95% confidence intervals are denoted. These charts are drawn from the data in Figures 1, 3 and 4.

Total BCAA showed a positive correlation with gastrocnemius muscle weight (Figure 6a), protein expression of LAT1 (Figure 6b), and BCKD activity (Figure 6c). Positive correlations were also observed between muscle weight and LAT1 (Figure 6d) and BCKD activity (Figure 6e).

## 4 DISCUSSION

While previous studies have examined the effect of cancer and/or chemotherapy on muscle mass and protein turnover (Ballarò et al., 2019; Barreto, Mandili, et al., 2016; Barreto, Waning, et al., 2016; Mora & Adegoke, 2021; Pin et al., 2019), we report, to our knowledge for the first time, sex differences in circulating and tissue BCAA availability and muscle BCAA transporter expression following administration of antineoplastic drugs to mice (Table 1). We show that body and gastrocnemius muscle weight losses in response to chemotherapy were more severe in female compared to male, consistent with greater loss of mTORC1/S6K1 signaling in muscles of female. However, drug‐treated male mice developed impaired insulin tolerance and experienced more severe decreases in skeletal muscle BCAA concentrations. The observed sex differences in BCAA levels might not be due to changes in LAT1, an established BCAA transporter, because treatment‐induced suppression of LAT1 expression was surprisingly more pronounced in females. Collectively, these data reveal previously undocumented sex‐and tissue‐specific alterations to muscle BCAA metabolism in response to chemotherapy and suggest that these changes may predict severity of chemotherapy‐related cachexia.

**TABLE 1:** Summary of main findings of drug treatment and sex.

Measured	Percent change (vehicle vs. drug)		*p*‐value of sex difference
Variable			
	Male	Female	
Body weight loss (Week 6)	10%^a^	15%^a^	0.0092^b^
Skeletal muscle weight			
Gastrocnemius	**13%** ^a^	**22%** ^a^	0.0371^b^
Tibialis anterior	**31%** ^a^	**34%** ^a^	0.8396
Quadriceps	**18%** ^a^	**11%** ^a^	0.2705
Insulin signaling			
p‐AKT^ser473^	**48%** ^a^	**44%** ^a^	0.8331
Anabolic signaling			
p‐S6K1^Thr389^	**36%** ^a^	**61%** ^a^	0.0492^b^
p‐S6^ser235/236^	**51%** ^a^	**38%** ^a^	0.2983
Protein synthesis	**71%** ^a^	**83%** ^a^	0.5744
Catabolic signaling			
p‐FOXO^ser256^	**56%**	**47%** ^a^	–
MuRF1	**109%**	**583%** ^a^	–
SM BCAA concentrations			
Leucine	**53%** ^a^	**32%** ^a^	0.0332^b^
Isoleucine	**59%** ^a^	**41%** ^a^	0.0232^b^
Valine	**74%** ^a^	**27%** ^a^	0.0004^b^
SM BCAA transporter			
LAT1	**40%** ^a^	**50%** ^a^	0.0264^b^
SM BCAA catabolism			
BCKD‐E1α	–	**56%** ^a^	–
p‐BCKD‐E1α^ser293^	**125%** ^a^	**113%** ^a^	0.2087
BCKD activity	**68%** ^a^	**51%** ^a^	0.3970
Plasma BCAA			
Leucine	**54%** ^a^	–	–
Valine	**57%** ^a^	–	–
Liver BCAA			
Leucine	**113%** ^a^	**66%** ^a^	0.1796
Valine	**147%** ^a^	**26%** ^a^	0.0495^b^

Abbreviations: BCAA, branched‐chain amino acids; BCKD, branched‐chain α‐keto acid dehydrogenase complex; p, phosphorylated; SM, skeletal muscle.

^a^ Indicates significant (*p* < 0.05) decrease (red) or increase (green) in sex (i.e., male vehicle vs. male drug, or female vehicle vs. female drug).

^b^ Indicates significant delta difference between sexes (i.e., male drug vs. female drug).

Identifying interventions that can prevent/mitigate cachexia has remained a holy grail in the field. For example, while the muscle anabolic effects of BCAA are incontrovertible, administration of these AAs has yielded minimal benefit on cachexia in humans (Harima et al., 2010; Pimentel et al., 2021; Poon et al., 2004; Soares et al., 2020). The anti‐anabolic and pro‐catabolic milieu that prevails in cachectic patients and rodents, as indicated by elevated circulating levels of catabolic factors such as tumor necrosis factor, IL‐6, and interferon gamma (de Matos‐Neto et al., 2015), are likely contributory to the recalcitrance to nutrients in cachectic conditions. Our data from cultured myotubes (Mora & Adegoke, 2024) and the in‐vivo data presented here clearly demonstrate that the blunted response to nutrients might be due to limitations in substrate delivery to muscle and/or accelerated efflux from skeletal muscle to plasma.

One of the main findings of this work is the sex differences in some of the commonly measured indicators of cachexia (body and tissue weights). In this study, drug‐treated female mice lost more body and gastrocnemius muscle weights compared to males. This is an important finding, given that skeletal muscle mass is a determinant of chemotherapy dose (Ali et al., 2016) and treatment effectiveness. In healthy subjects, males have greater muscle mass than females (Janssen et al., 2000) and higher levels of testosterone, the anabolic hormone that enhances muscle mass (Bhasin et al., 2001). In addition, females have lower blood elimination (Kim et al., 2018) and drug clearance (Wagner et al., 2019) for chemotherapy agents, supporting a more catabolic environment in females. Therefore, females may experience more side effects and decreased chemotherapy effectiveness compared to males. Since chemotherapy can also reduce fat mass (Ebadi et al., 2017), we were surprised to see no significant effect of chemotherapy on the adipose tissue weight in both sexes, especially since females have a greater fat mass compared to males (Karastergiou et al., 2012). However, it is important to note that cachexia can occur with or without the loss of adipose tissue (Ni & Zhang, 2020) and since these animals are relatively young in age, it is possible they do not have large fat storage, compared to the fat storage seen in aging (Ponti et al., 2020).

The differences observed between the sexes may be related to sex‐dependent alterations in insulin sensitivity. In myotubes, we have shown that chemotherapy leads to decreased insulin‐stimulated glucose uptake (Mora & Adegoke, 2021). Our finding of worsened insulin sensitivity only in males is likely related to hormonal differences between the sexes, as estrogen, a predominately female hormone, has protective effects against insulin resistance (De Paoli et al., 2021). Therefore, repeating these experiments in aged post‐menopausal female animals may produce different results.

The observed sex‐differences in S6K1 phosphorylation are consistent with the reduced muscle weight observed in females. In healthy subjects, minimal sex differences exist for mTORC1 signaling and protein synthesis (Dreyer et al., 2010; West et al., 2012). However, we found that in healthy mice administered chemotherapy, the reduction in mTORC1 signaling was more severe in drug‐treated female animals compared to males. Also, drug‐treated female mice had increased MuRF1 protein expression, a finding not seen in males. This is contrary to previous literature, whereby males have greater ubiquitin proteasome activity compared to females (Ogawa et al., 2017). However, we only measured protein expression of an E3 ligase, but not proteasome activity per se. Our findings indicate that in healthy mice, administration of chemotherapy led to sex differences in mechanisms related to protein synthesis/breakdown, which may contribute at least in part to the more severe loss of gastrocnemius muscle weight in drug‐treated female mice.

Another main finding of this work is the profound sex differences in tissue and circulating BCAA levels. All 20 AAs are required for protein synthesis in skeletal muscle (Wolfe, 2017). Males have higher muscle concentrations of the BCAA (Costanzo et al., 2022). Therefore, higher BCAA concentrations may present a protective effect against chemotherapy‐induced cachexia in males, an observation that may partially explain the more severe loss of gastrocnemius muscle weight in females. More severe reductions in muscle BCAA concentrations in males could simply be due to the greater BCAA pools found in males (Costanzo et al., 2022), giving these animals more to lose. However, during skeletal muscle substrate deficits, males tend to oxidize more AAs for energy, while females tend to oxidize more fats (Lamont et al., 2001). Therefore, following chemotherapy, males may generate more energy from muscle AAs to support protein synthesis and abrogate some of the loss of muscle mass from chemotherapy, an effect that is further supported by reduced arginine concentrations seen only in males. Decreased concentrations of BCAA in both sexes is associated with decreased expression of their transporter LAT1, an effect that is more severe in females. The greater loss of LAT1 in females may be explained by the fact that LAT1 can function as a bi‐directional transporter (Kahlhofer & Teis, 2022). Female animals lost more muscle, had greater S6K1 suppression, higher MuRF1 expression and greater ubiquitination of proteins, all consistent with a more catabolic environment in females. Therefore, the greater reduction in LAT1 in females may represent an adaptation to limit the loss of BCAA and other AAs in females, thus limiting atrophy.

Decreased BCAA concentrations in the skeletal muscle following chemotherapy may also be related to altered metabolism of these AAs. The key enzymes involved in BCAA catabolism, mainly BCAT1/2 and BCKD, are elevated in tumors of the liver (Ericksen et al., 2019) and breast (Zhang & Han, 2017). However, these enzymes are not typically studied in the skeletal muscle of cancer‐inoculated mice, nor following chemotherapy. It is possible that the decreased BCKD activity in the skeletal muscle of both sexes following chemotherapy is a compensatory mechanism to preserve the BCAA pool.

Lastly, it is interesting to note tissue differences in BCAA handling during chemotherapy. The increase in plasma BCAA in males can be related to the release of these AAs from skeletal muscle, a tissue that may rely on the oxidation of AAs during substrate deficits (Lamont et al., 2001). Also, a previous review has outlined that dysregulation of BCAA metabolism in the gut microbiome can result in elevated plasma BCAA (Li et al., 2023), an interesting finding as chemotherapy may cause changes in gut microbiome (Wu et al., 2022). However, we did not measure gut microbiome in this study.

Our data on liver BCAA and LAT1 expression in both sexes indicates that the liver is the main receiver of muscle‐derived BCAA. In the liver, AAs such as alanine and glutamine (Gannon & Nuttall, 2010), as well valine (Mann et al., 2021), which was found to be higher following drug treatment, can be converted into glucose by gluconeogenesis. Therefore, it is likely that the increase in liver BCAA is a compensatory mechanism in an attempt to not only remove excess BCAA from the plasma, but to also generate glucose at a time following chemotherapy when there are substrate deficits.

A limitation of this study is that glucose levels during our ITT could be confounded by chemotherapy‐induced changes in baseline glucose. However, we observed no significant treatment effects on the slopes of the glucose curves during ITTs. Another limitation is that in real life, chemotherapy drugs are not given to healthy individuals. However, we have used clinically relevant chemotherapy drugs (Tabernero et al., 2015) to probe sex differences in metabolic responses, especially BCAA catabolism, to these drugs. The fact that elevations in liver BCAA concentrations (Ericksen et al., 2019; Zhang & Han, 2017) are seen in many cancers is consistent with observations made in this study, and suggests that the changes we observed will likely be heightened when tumor implantation is combined with chemotherapy, a subject that our lab is interested in. Lastly, we did not include interventional treatments here. However, since sex differences in BCAA metabolism following chemotherapy are rarely studied, it was difficult to decide on appropriate interventions. Findings from this study offer possibilities for designing future interventional studies.

In conclusion, compared to male animals, drug‐treated female animals experienced worsened outcomes for body and gastrocnemius muscle weights, and p‐S6K1^thr389^ signaling. On the other hand, drug‐treated male animals were more insulin resistant and experienced more severe decreases in skeletal muscle BCAA concentrations. We did not directly study whether the altered BCAA metabolism has a causative effect. However, the positive correlations that we observed between both LAT1 and BCKD activity and muscle weight suggest that disruption of BCAA metabolism may play a role in cachexia and that interventions that can correct the disruption may help in the management of this condition.

## AUTHOR CONTRIBUTIONS

SM and OAJA designed the experiments. SM and GM performed the experiments. SM analyzed the samples and drafted the initial version of the manuscript. OAJA reviewed and edited the manuscript. SM, GM and OAJA approve the final version of the manuscript.

## FUNDING INFORMATION

This study was funded by grants from the Natural Science and Engineering Research Council of Canada (NSERC RGPIN‐2021‐03603) and from the Faculty of Health, York University, Toronto Canada to OAJA.

## CONFLICT OF INTEREST STATEMENT

The authors declare no conflicts of interest.

## ETHICS STATEMENT

All animal experiments were approved by the York University Animal Care Committee in accordance with the recommendations of the Canadian Council on Animal Care.

## Supporting information


Data S1.

## Data Availability

The data that support the findings of this study are available from the corresponding author upon reasonable request.

## References

[phy216003-bib-0001] Adegoke, O. A. J. , Abdullahi, A. , & Tavajohi‐Fini, P. (2012). MTORC1 and the regulation of skeletal muscle anabolism and mass. Applied Physiology, Nutrition and Metabolism, 37, 395–406. 10.1139/h2012-009 22509811

[phy216003-bib-0002] Ali, R. , Baracos, V. E. , Sawyer, M. B. , Bianchi, L. , Roberts, S. , Assenat, E. , Mollevi, C. , & Senesse, P. (2016). Lean body mass as an independent determinant of dose‐limiting toxicity and neuropathy in patients with colon cancer treated with FOLFOX regimens. Cancer Medicine, 5, 607–616. 26814378 10.1002/cam4.621 PMC4831278

[phy216003-bib-0003] Ballarò, R. , Beltrà, M. , De Lucia, S. , Pin, F. , Ranjbar, K. , Hulmi, J. J. , Costelli, P. , & Penna, F. (2019). Moderate exercise in mice improves cancer plus chemotherapy‐induced muscle wasting and mitochondrial alterations. FASEB Journal, 33, 5482–5494. 30653354 10.1096/fj.201801862R

[phy216003-bib-0004] Barreto, R. , Mandili, G. , Witzmann, F. A. , Novelli, F. , Zimmers, T. A. , & Bonetto, A. (2016). Cancer and chemotherapy contribute to muscle loss by activating common signaling pathways. Frontiers in Physiology, 7, 472. 10.3389/fphys.2016.00472 27807421 PMC5070123

[phy216003-bib-0005] Barreto, R. , Waning, D. L. , Gao, H. , Liu, Y. , Zimmers, T. A. , & Bonetto, A. (2016). Chemotherapy‐related cachexia is associated with mitochondrial depletion and the activation of ERK1/2 and p38 MAPKs. Oncotarget, 7, 43442–43460. 27259276 10.18632/oncotarget.9779 PMC5190036

[phy216003-bib-0006] Bhasin, S. , Woodhouse, L. , Casaburi, R. , Singh, A. B. , Bhasin, D. , Berman, N. , Chen, X. , Yarasheski, K. E. , Magliano, L. , Dzekov, C. , Dzekov, J. , Bross, R. , Phillips, J. , Sinha‐Hikim, I. , Shen, R. , & Storer, T. W. (2001). Testosterone dose‐response relationships in healthy young men. American Journal of Physiology. Endocrinology and Metabolism, 281, E1172–E1181. 11701431 10.1152/ajpendo.2001.281.6.E1172

[phy216003-bib-0007] Chantranupong, L. , Wolfson, R. L. , Orozco, J. M. , Saxton, R. A. , Scaria, S. M. , Bar‐Peled, L. , Spooner, E. , Isasa, M. , Gygi, S. P. , & Sabatini, D. M. (2014). The sestrins interact with gator2 to negatively regulate the amino‐acid‐sensing pathway upstream of mTORC1. Cell Reports, 9, 1–8. 25263562 10.1016/j.celrep.2014.09.014 PMC4223866

[phy216003-bib-0008] Costanzo, M. , Caterino, M. , Sotgiu, G. , Ruoppolo, M. , Franconi, F. , & Campesi, I. (2022). Sex differences in the human metabolome. Biology of Sex Differences, 13, 30. 10.1186/s13293-022-00440-4 35706042 PMC9199320

[phy216003-bib-0009] de Matos‐Neto, E. M. , Lima, J. D. C. C. , de Pereira, W. O. , Riccardi, D. M. d. R. , Radloff, K. , das Neves, R. X. , Camargo, R. G. , Maximiano, L. F. , Tokeshi, F. , Otoch, J. P. , Goldszmid, R. , Câmara, N. O. S. , Trinchieri, G. , de Alcântara, P. S. M. , & Seelaender, M. (2015). Systemic inflammation in cachexia–Is tumor cytokine expression profile the culprit? Frontiers in Immunology, 6, 629. 26732354 10.3389/fimmu.2015.00629 PMC4689790

[phy216003-bib-0010] De Paoli, M. , Zakharia, A. , & Werstuck, G. H. (2021). The role of estrogen in insulin resistance. The American Journal of Pathology, 191, 1490–1498. 34102108 10.1016/j.ajpath.2021.05.011

[phy216003-bib-0011] Dreyer, H. C. , Fujita, S. , Glynn, E. L. , Drummond, M. J. , Volpi, E. , & Rasmussen, B. B. (2010). Resistance exercise increases leg muscle protein synthesis and mTOR signalling independent of sex. Acta Physiologica, 199, 71–81. 20070283 10.1111/j.1748-1716.2010.02074.x PMC2881180

[phy216003-bib-0012] Ebadi, M. , Field, C. J. , Lehner, R. , & Mazurak, V. C. (2017). Chemotherapy diminishes lipid storage capacity of adipose tissue in a preclinical model of colon cancer. Lipids in Health and Disease, 16, 247. 29258509 10.1186/s12944-017-0638-8 PMC5735884

[phy216003-bib-0013] Ericksen, R. E. , Lim, S. L. , McDonnell, E. , Shuen, W. H. , Vadiveloo, M. , White, P. J. , Ding, Z. , Kwok, R. , Lee, P. , Radda, G. K. , Toh, H. C. , Hirschey, M. D. , & Han, W. (2019). Loss of BCAA catabolism during carcinogenesis enhances mTORC1 activity and promotes tumor development and progression. Cell Metabolism, 29, 1151–1165. 10.1016/j.cmet.2018.12.020 30661928 PMC6506390

[phy216003-bib-0014] Evans, W. J. , Morley, J. E. , Argilés, J. , Bales, C. , Baracos, V. , Guttridge, D. , Jatoi, A. , Kalantar‐Zadeh, K. , Lochs, H. , Mantovani, G. , Marks, D. , Mitch, W. E. , Muscaritoli, M. , Najand, A. , Ponikowski, P. , Rossi Fanelli, F. , Schambelan, M. , Schols, A. , Schuster, M. , … Anker, S. D. (2008). Cachexia: A new definition. Clinical Nutrition, 27, 793–799. 18718696 10.1016/j.clnu.2008.06.013

[phy216003-bib-0015] Gannon, M. C. , & Nuttall, F. Q. (2010). Amino acid ingestion and glucose metabolism‐a review. IUBMB Life, 62, 660–668. 20882645 10.1002/iub.375

[phy216003-bib-0016] Gomes‐Marcondes, M. C. C. , Ventrucci, G. , Toledo, M. T. , Cury, L. , & Cooper, J. C. (2003). A leucine‐supplemented diet improved protein content of skeletal muscle in young tumor‐bearing rats. Brazilian Journal of Medical and Biological Research, 36, 1589–1594. 10.1590/S0100-879X2003001100017 14576914

[phy216003-bib-0017] Harima, Y. , Yamasaki, T. , Hamabe, S. , Saeki, I. , Okita, K. , Terai, S. , & Sakaida, I. (2010). Effect of a late evening snack using branched‐chain amino acid‐enriched nutrients in patients undergoing hepatic arterial infusion chemotherapy for advanced hepatocellular carcinoma. Hepatology Research, 40, 574–584. 20618455 10.1111/j.1872-034X.2010.00665.x

[phy216003-bib-0018] Janssen, I. , Heymsfield, S. B. , Wang, Z. , & Ross, R. (2000). Skeletal muscle mass and distribution in 468 men and women aged 18–88 yr. Journal of Applied Physiology, 89, 81–88. 10904038 10.1152/jappl.2000.89.1.81

[phy216003-bib-0019] Jeganathan, S. , Abdullahi, A. , Zargar, S. , Maeda, N. , Riddell, M. C. , & Adegoke, O. A. J. (2014). Amino acid‐induced impairment of insulin sensitivity in healthy and obese rats is reversible. Physiological Reports, 2, e12067. 24997070 10.14814/phy2.12067 PMC4187556

[phy216003-bib-0020] Kahlhofer, J. , & Teis, D. (2022). The human LAT1–4F2hc (SLC7A5–SLC3A2) transporter complex: Physiological and pathophysiological implications. Basic & Clinical Pharmacology & Toxicology, 133, 459–472. 36460306 10.1111/bcpt.13821 PMC11497297

[phy216003-bib-0021] Karastergiou, K. , Smith, S. R. , Greenberg, A. S. , & Fried, S. K. (2012). Sex differences in human adipose tissues–The biology of pear shape. Biology of Sex Differences, 3, 13. 22651247 10.1186/2042-6410-3-13 PMC3411490

[phy216003-bib-0022] Kim, H. I. , Lim, H. , & Moon, A. (2018). Sex differences in cancer: Epidemiology, genetics and therapy. Biomolecules & Therapeutics, 26, 335–342. 29949843 10.4062/biomolther.2018.103 PMC6029678

[phy216003-bib-0023] Lamont, L. S. , McCullough, A. J. , & Kalhan, S. C. (2001). Gender differences in leucine, but not lysine, kinetics. Journal of Applied Physiology, 91, 357–362. 11408452 10.1152/jappl.2001.91.1.357

[phy216003-bib-0024] Li, N. , Cen, Z. , Zhao, Z. , Li, Z. , & Chen, S. (2023). BCAA dysmetabolism in the host and gut microbiome, a key player in the development of obesity and T2DM. Medicine in Microecology, 16, 100078.

[phy216003-bib-0025] Lim, S. , Brown, J. L. , Washington, T. A. , & Greene, N. P. (2020). Development and progression of cancer cachexia: Perspectives from bench to bedside. Sports Medicine and Health Science, 2, 177–185. 34447946 10.1016/j.smhs.2020.10.003 PMC8386816

[phy216003-bib-0026] Mann, G. , & Adegoke, O. A. J. (2021). Effects of ketoisocaproic acid and inflammation on glucose transport in muscle cells. Physiological Reports, 9, e14673. 33400857 10.14814/phy2.14673 PMC7785050

[phy216003-bib-0027] Mann, G. , Mora, S. , Madu, G. , & Adegoke, O. A. J. (2021). Branched‐chain amino acids: Catabolism in skeletal muscle and implications for muscle and whole‐body metabolism. Frontiers in Physiology, 12, 702826. 34354601 10.3389/fphys.2021.702826 PMC8329528

[phy216003-bib-0028] Mittendorfer, B. , Horowitz, J. F. , & Klein, S. (2002). Effect of gender on lipid kinetics during endurance exercise of moderate intensity in untrained subjects. American Journal of Physiology. Endocrinology and Metabolism, 283, E58–E65. 10.1152/ajpendo.00504.2001 12067843

[phy216003-bib-0029] Mora, S. , & Adegoke, O. A. J. (2021). The effect of a chemotherapy drug cocktail on myotube morphology, myofibrillar protein abundance, and substrate availability. Physiological Reports, 9, e14927. 34197700 10.14814/phy2.14927 PMC8248921

[phy216003-bib-0030] Mora, S. , & Adegoke, O. A. J. (2024). Maintenance of the branched‐chain amino acid transporter LAT1 counteracts myotube atrophy following chemotherapy. American Journal of Physiology. Cell Physiology, 326, C866–C879. 38284122 10.1152/ajpcell.00537.2023

[phy216003-bib-0031] Ni, J. , & Zhang, L. (2020). Cancer cachexia: Definition, staging, and emerging treatments. Cancer Management and Research, 12, 5597–5605. 10.2147/CMAR.S261585 32753972 PMC7358070

[phy216003-bib-0032] Obayashi, M. , Shimomura, Y. , Nakai, N. , Jeoung, N. H. , Nagasaki, M. , Murakami, T. , Sato, Y. , & Harris, R. A. (2004). Estrogen controls branched‐chain amino acid catabolism in female rats. Journal of Nutrition, 134, 2628–2633. 15465758 10.1093/jn/134.10.2628

[phy216003-bib-0033] Ogawa, M. , Kitano, T. , Kawata, N. , Sugihira, T. , Kitakaze, T. , Harada, N. , & Yamaji, R. (2017). Daidzein down‐regulates ubiquitin‐specific protease 19 expression through estrogen receptor β and increases skeletal muscle mass in young female mice. The Journal of Nutritional Biochemistry, 49, 63–70. 28886438 10.1016/j.jnutbio.2017.07.017

[phy216003-bib-0034] Pimentel, G. D. , Pichard, C. , Laviano, A. , & Fernandes, R. C. (2021). High protein diet improves the overall survival in older adults with advanced gastrointestinal cancer. Clinical Nutrition, 40, 1376–1380. 10.1016/j.clnu.2020.08.028 32919817

[phy216003-bib-0035] Pin, F. , Barreto, R. , Couch, M. E. , Bonetto, A. , & O'Connell, T. M. (2019). Cachexia induced by cancer and chemotherapy yield distinct perturbations to energy metabolism. Journal of Cachexia, Sarcopenia and Muscle, 10, 140–154. 10.1002/jcsm.12360 30680954 PMC6438345

[phy216003-bib-0036] Ponti, F. , Santoro, A. , Mercatelli, D. , Gasperini, C. , Conte, M. , Martucci, M. , Sangiorgi, L. , Franceschi, C. , & Bazzocchi, A. (2020). Aging and imaging assessment of body composition: From fat to facts. Front Endocrinol (Lausanne), 10, 861. 31993018 10.3389/fendo.2019.00861 PMC6970947

[phy216003-bib-0037] Poon, R. T.‐P. , Yu, W.‐C. , Fan, S.‐T. , & Wong, J. (2004). Long‐term oral branched chain amino acids in patients undergoing chemoembolization for hepatocellular carcinoma: A randomized trial. Alimentary Pharmacology & Therapeutics, 19, 779–788. 15043519 10.1111/j.1365-2036.2004.01920.x

[phy216003-bib-0038] Rosa‐Caldwell, M. E. , & Greene, N. P. (2019). Muscle metabolism and atrophy: Let's talk about sex. Biology of Sex Differences, 10, 43. 31462271 10.1186/s13293-019-0257-3 PMC6714453

[phy216003-bib-0039] Soares, J. D. P. , Siqueira, J. M. , Oliveira, I. C. L. , Laviano, A. , & Pimentel, G. D. (2020). A high‐protein diet, not isolated BCAA, is associated with skeletal muscle mass index in patients with gastrointestinal cancer. Nutrition, 72, 110698. 10.1016/j.nut.2019.110698 32007808

[phy216003-bib-0040] Tabernero, J. , Yoshino, T. , Cohn, A. L. , Obermannova, R. , Bodoky, G. , Garcia‐Carbonero, R. , Ciuleanu, T.‐E. , Portnoy, D. C. , Van Cutsem, E. , Grothey, A. , Prausová, J. , Garcia‐Alfonso, P. , Yamazaki, K. , Clingan, P. R. , Lonardi, S. , Kim, T. W. , Simms, L. , Chang, S.‐C. , & Nasroulah, F. (2015). Ramucirumab versus placebo in combination with second‐line FOLFIRI in patients with metastatic colorectal carcinoma that progressed during or after first‐line therapy with bevacizumab, oxaliplatin, and a fluoropyrimidine (RAISE): A randomised, double‐blind, multicentre, phase 3 study. The Lancet Oncology, 16, 499–508. 25877855 10.1016/S1470-2045(15)70127-0

[phy216003-bib-0041] Tisdale, M. J. (2005). Molecular pathways leading to cancer cachexia. Physiology, 20, 340–349. 16174873 10.1152/physiol.00019.2005

[phy216003-bib-0042] Ventrucci, G. , Ramos Silva, L. G. , Roston Mello, M. A. , & Gomes Marcondes, M. C. C. (2004). Effects of a leucine‐rich diet on body composition during nutritional recovery in rats. Nutrition, 20, 213–217. 10.1016/j.nut.2003.10.014 14962689

[phy216003-bib-0043] Wagner, A. D. , Oertelt‐Prigione, S. , Adjei, A. , Buclin, T. , Cristina, V. , Csajka, C. , Coukos, G. , Dafni, U. , Dotto, G. P. , Ducreux, M. , Fellay, J. , Haanen, J. , Hocquelet, A. , Klinge, I. , Lemmens, V. , Letsch, A. , Mauer, M. , Moehler, M. , Peters, S. , & Özdemir, B. C. (2019). Gender medicine and oncology: Report and consensus of an ESMO workshop. Annals of Oncology, 30, 1914–1924. 31613312 10.1093/annonc/mdz414

[phy216003-bib-0044] West, D. W. D. , Burd, N. A. , Churchward‐Venne, T. A. , Camera, D. M. , Mitchell, C. J. , Baker, S. K. , Hawley, J. A. , Coffey, V. G. , & Phillips, S. M. (2012). Sex‐based comparisons of myofibrillar protein synthesis after resistance exercise in the fed state. Journal of Applied Physiology, 112, 1805–1813. 22383503 10.1152/japplphysiol.00170.2012

[phy216003-bib-0045] White, P. J. , McGarrah, R. W. , Grimsrud, P. A. , Tso, S. C. , Yang, W. H. , Haldeman, J. M. , Grenier‐Larouche, T. , An, J. , Lapworth, A. L. , Astapova, I. , Hannou, S. A. , George, T. , Arlotto, M. , Olson, L. B. , Lai, M. , Zhang, G. F. , Ilkayeva, O. , Herman, M. A. , Wynn, R. M. , … Newgard, C. B. (2018). The BCKDH kinase and phosphatase integrate BCAA and lipid metabolism via regulation of ATP‐citrate Lyase. Cell Metabolism, 27, 1281–1293. 29779826 10.1016/j.cmet.2018.04.015 PMC5990471

[phy216003-bib-0046] Wolfe, R. R. (2017). Branched‐chain amino acids and muscle protein synthesis in humans: Myth or reality? Journal of the International Society of Sports Nutrition, 14, 30. 28852372 10.1186/s12970-017-0184-9 PMC5568273

[phy216003-bib-0047] Wu, A. H. , Vigen, C. , Tseng, C. , Garcia, A. A. , & Spicer, D. (2022). Effect of chemotherapy on the gut microbiome of breast cancer patients during the first year of treatment. Breast Cancer: Targets and Therapy, 14, 433–451. 36532254 10.2147/BCTT.S305486 PMC9747861

[phy216003-bib-0048] Zargar, S. , Moreira, T. S. , Samimi‐Seisan, H. , Jeganathan, S. , Kakade, D. , Islam, N. , Campbell, J. , & Adegoke, O. A. J. (2011). Skeletal muscle protein synthesis and the abundance of the mRNA translation initiation repressor PDCD4 are inversely regulated by fasting and refeeding in rats. American Journal of Physiology. Endocrinology and Metabolism, 300, E986–E992. 21406616 10.1152/ajpendo.00642.2010

[phy216003-bib-0049] Zhang, L. , & Han, J. (2017). Branched‐chain amino acid transaminase 1 (BCAT1) promotes the growth of breast cancer cells through improving mTOR‐mediated mitochondrial biogenesis and function. Biochemical and Biophysical Research Communications, 486, 224–231. 28235484 10.1016/j.bbrc.2017.02.101

