# Supplementary material for: Sex differences in cachexia and branched‐chain amino acid metabolism following chemotherapy in mice
Source: Physiol Rep. 2024 Apr 17;12(8):e16003. doi: 10.14814/phy2.16003 (PMC11023815; doi:10.14814/phy2.16003)
Supplement: Supplementary file 1 — Data S1. [file PHY2-12-e16003-s001.docx]

**Supplementary figures for “Sex Differences in Cachexia and Branched-Chain Amino Acid Metabolism Following Chemotherapy in Mice”**

Stephen Mora, Gagandeep Mann, and Olasunkanmi A J Adegoke

**Supplementary Table I – Primary Antibody, Source and Company (Cat#)**

| **ANTIBODY** | **SOURCE** | **COMPANY** |
| --- | --- | --- |
| MHC-1 | Mouse | Developmental Hybridoma (MF-20) |
| Troponin | Mouse | Developmental Hybridoma (JLT12) |
| Tropomyosin | Mouse | Developmental Hybridoma (CH-1) |
| p-FoxO3a^ser253^ | Rabbit | Cell Signalling Tech (#9466) |
| p-AKT^ser473^ | Rabbit | Cell Signalling Tech (#4060) |
| p-S6^ser235/236^ | Rabbit | Cell Signalling Tech (#4858) |
| p-S6K1^thr389^ | Rabbit | Cell Signalling Tech (#9234) |
| p-4E-BP1^thr37/46^ | Rabbit | Cell Signalling Tech (#2855) |
| SNAT1 | Rabbit | Cell Signalling Tech (#36057) |
| p-BCKD-E1α^ser293^ | Rabbit | Cell Signalling Tech (#40368) |
| BCKD-E1α | Rabbit | Cell Signalling Tech (#90198) |
| BCAT2 | Rabbit | Protein Tech (#16417-1-AP) |
| MuRF1 | Rabbit | Protein Tech (#55456-1-AP) |
| BDK | Rabbit | Invitrogen (#PA5-31455) |
| LAT1 | Rabbit | Invitrogen (#PA5-50485) |
| γ-tubulin | Mouse | Sigma Aldrich (#T6557) |
| Puromycin | Mouse | EMD Millipore (#MABE343) |
| Ubiquitin | Mouse | Santa Cruz (#sc-8017) |

**Supplementary Table II – Raw Body and Tissue Weights in Animals**

| **Body/Tissue** | **Male Vehicle** | **Female Vehicle** |
| --- | --- | --- |
| Initial Body Weight | 28.3±3.1 | 22.6±0.9 |
| Final Body Weight | 29.4±3.1 | 25.6±2 |
| Gastrocnemius | 190±40.6 | 166±34.1 |
| Tibialis Anterior | 57±10.8 | 39±6.4 |
| Quadriceps | 193±35.4 | 162±19.9 |
| Visceral Adipose | 250±95.6 | 278±118 |
| Kidney | 271±31.7 | 165±24.2 |
| Liver | 1193±135.7 | 1003±131.3 |
| Spleen | 106±21.8 | 126±20.4 |
| **Body/Tissue** | **Male Drug** | **Female Drug** |
| Initial Body Weight | 28.8±2.3 | 24.3±1.5 |
| Final Body Weight | 25.7±1.8 | 20.6±1.4 |
| Gastrocnemius | 146±21.5 | 113±11.4 |
| Tibialis Anterior | 37±7.8 | 21.6±3.7 |
| Quadriceps | 151±38.6 | 113±16 |
| Visceral Adipose | 224±87.6 | 192.6±76.9 |
| Kidney | 254±33.1 | 153±22.9 |
| Liver | 1110±130.7 | 1006±179 |
| Spleen | 266±184 | 207±33.2 |

Body weights in g; raw tissue weights are in mg; data are Mean±SD.


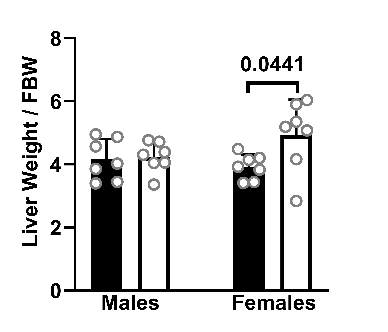

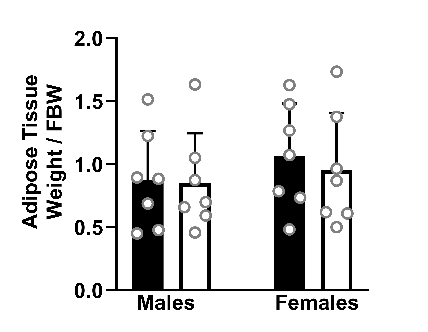

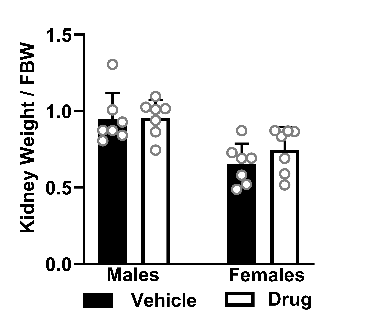

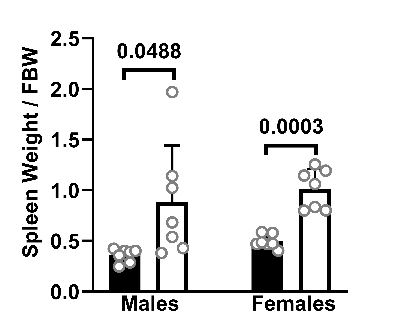


**B**

**A**

**D**

**C**

**MHC-1**

**-250kDa**

**γ-tubulin**

**-50kDa**

**D**

**V**

**D**

**V**

**Troponin**

**-37kDa**

**-50kDa**

**γ-tubulin**

**Tropomyosin**

**-37kDa**

**-50kDa**


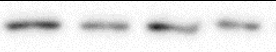

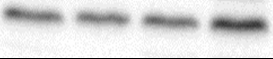


**γ-tubulin**


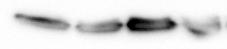

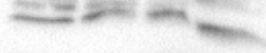


**Males**

**Females**


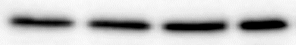

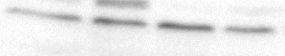

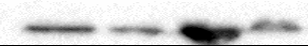

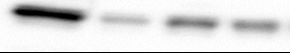

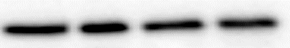

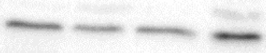

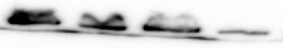

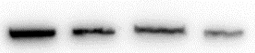


**D**

**V**

**D**

**V**

**E**


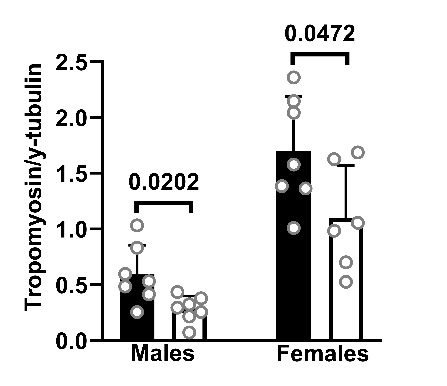

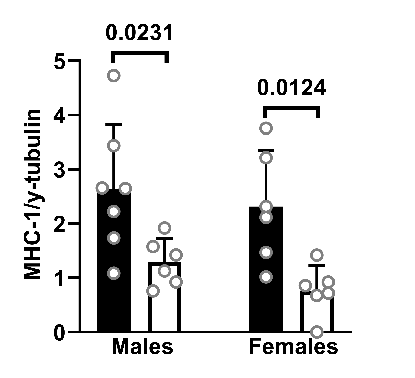

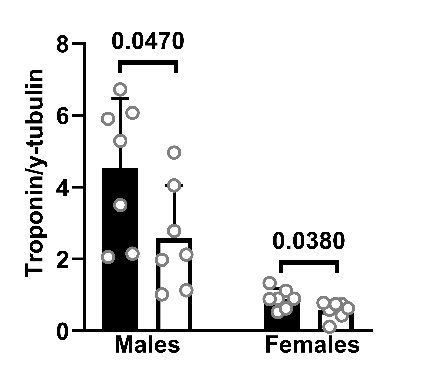


**H**

**G**

**F**

**Supplementary Figure 1, related to Manuscript Figure 1. Effects of chemotherapy drug treatment on organ weights and myofibrillar protein content.** Male and female 10-12-week-old CD2F1 mice were treated with either vehicle (V, 3.8% DMSO in saline, black bars) or a chemotherapy drug cocktail (D, Drug: 50mg/kg 5-FLU, 90mg/kg Leucovorin, 24mg/kg CPT11, white bars) twice a week for 6 weeks. Mice were euthanized at least 24h after their last chemotherapy dose. No significant effects of drug treatment on kidney **(A)** and visceral white adipose tissue **(B)** weights were found, but spleen weight was higher in both sexes **(C)**. Only drug-treated females showed an increase in liver weight **(D)**. Irrespective of sex, drug treatment reduced the expression of myosin heavy chain-1 (MHC-1) **(E, F)**, troponin **(E, G)** and tropomyosin **(E, H)** compared to controls in the gastrocnemius muscle. Data are mean ± SD; n = 6-7 animals per group.

Leucine Isoleucine Valine

KIC KMV KIV

BCAT2

BCKD

**Isovaleryl-CoA**

**2-methylbutyryl-CoA**

**Isobutyryl-CoA**

**Supplementary Figure 2, related to Manuscript Figure 4. A simplified diagram of the first two steps in BCAA catabolism is shown.** The BCAA are first reversibly transaminated by BCAT2, forming the BCKA. The BCKA are then irreversibly oxidatively decarboxylated by BCKD to their respective acyl-CoA derivatives. BCAT2, Branched-chain aminotransferase; BCKD, Branched-chain α-keto acid dehydrogenase complex; KIC, 2-keto-isocaproate/4-methyl-2-oxopentanoic acid; KMV, α-keto-β-methylvaleric acid/3-methyl-2-oxopentanoate; KIV, 2-keto-isovalerate/3-methyl-2-oxobutanoic acid. BCKA, branched-chain α-keto acids.
